# The Common Task

**Published:** 1907-08-10

**Authors:** 


### THE COMMON TASK.

**Correspondence and Queries** for this section should be sent to the Editor of The Hospital, 28 Southampton Street, Strand, London, and marked "Nursing Administration."

### The Cost of Advertising.

The new movement in favour of economy in Poor-law institutions has roused the doubt whether it is necessary to expend such large sums as it is usual to do over advertising the minor appointments. A few weeks ago the porter's place became vacant in one of the Metropolitan infirmaries. The wages were about 24s. a week, and the sum expended on filling the post was £7. The result of this widespread announcement was to produce several hundred applications, and when the collective cost of postage is taken into consideration together with the disappointment incurred, it certainly seems as though the Guardians had been a little over-anxious in their endeavour to secure the best man in London to stand at their door. At the same time there is a great safeguard in insisting on the public announcement of posts, even of a minor character in public institutions, and the cheap plan of posting a notice on the gate, which would be adopted in a private business is not one which could be copied with any safety by a Board of Guardians.

### County Nursing Associations.

The formation of the Kent County Nursing Association is another important step in the direction of securing to the rural districts an efficient nursing system. Until there is a central nursing organisation for each county there can be no security for the care of the sick, for there can be no continuity in the isolated efforts made in various localities to establish nursing societies. Such local societies depend too often on the energy of one individual, and are apt to dwindle and die when personal zeal is withdrawn. Moreover, they are often in difficulties from an imperfect organisation, or from inexperience. In such cases the parent Association with command of wider resources can stimulate to fresh exertions, or remove friction by an authoritative word of advice. The support which it is able to enlist is essential to the development of midwifery in scattered districts, and we rejoice to see that this branch of the work is receiving full recognition at the hands of the Kent County Association.

### Wasted Vegetables.

What becomes of the vegetables which are left over when dinner is done? This is a question which some may deem to stand in the same category as the poet's cry, "Où sont les neiges d'automne?" The incinerator knows. The dustman knows. The pigs who fatten on the waste products of the hospital know. But the housekeeper is indifferent and the cook contemptuous. Whatever may be the case with meat which is twice cooked, vegetables undergo no deterioration by being warmed up. They may be used in several ways. Skilfully treated, they may in fact make a better show on their second appearance than under the usual insipid form to which English people cling. They may be re-heated by steaming, placed in a fireproof dish, and served with a parsley and butter sauce garnished with rolls of bacon, as a supper dish. Or with a little onion added they may be used for a vegetable curry, and served surrounded with boiled rice. Or they may be chopped small, covered with mashed potato, and browned in the oven as vegetable pie. Even the despised cold cabbage can be made into a tasty dish if fried with a little lean ham. These and other suggestions for "Dishes Made Without Meat" are to be found in Mrs. Peel's new shilling cookery book sold under this title (Constable and Co.), and cordially to be recommended for summer use.

### Bogus Nursing Homes.

Do these exist? In so far as by bogus homes is meant establishments owned and run by professional thieves, we must confess ourselves frankly sceptical. The expenses of running a home are considerable, and the profits from the point of view of the thief are next to nothing. It is hard enough to make a decent living out of a nursing home, even when a good connection has been formed. And the liabilities incurred are out of all proportion to the sum which could be made by pilfering from the patients. What woman in her senses would take her jewels to a nursing home? What man would take rouleaus of sovereigns with him? The more usual course is to pay by cheque either at the end of the term or weekly, and the risk of purloining articles of personal property is one which no professional thief would be disposed to incur. No. The bogus nursing home resolves into a bogey; but none the less the number of thoroughly unsatisfactory nursing homes is large enough to rouse a strong conviction in many quarters that they ought not to be allowed to continue. They commonly come into being on the initiation of some sanguine and inexperienced nurse who is tired of private nursing. She has probably pleasant manners, and can persuade doctors in her neighbourhood to entrust her with patients. She has no idea of household management, no money to equip her house decently, although she may produce a superficial appearance of elegance, and nothing to fall back upon during the slack times. She is exorbitant in her charges to those who can afford to pay, and cuts down the running expenses till her unhappy patients are frequently half-starved under the guise of treatment. She will not pay for good nurses for her patients, and is therefore a prey to any shady individuals who will come for low fees in consideration of not being cross-examined about testimonials. The house is dirty, the service is bad, the nursing is slovenly; yet such is the ignorance of the public about these matters that this kind of home drifts on for years without any scandal ensuing, unless one of the half-trained women employed decamps with a few rings, or credit failing altogether, the unhappy proprietress, in a vain struggle against fate, borrows her patient's purse in order to buy the dinner. That this state of things should exist is a discredit to the medical profession, who could at will entirely put an end to this class of establishment by refusing to recommend patients without satisfying themselves by personal inspection of the manner in which each home is conducted.

